# Dynamic Analysis of the Conditional Oscillator Underlying Slow Waves in Thalamocortical Neurons

**DOI:** 10.3389/fncir.2016.00010

**Published:** 2016-02-25

**Authors:** François David, Vincenzo Crunelli, Nathalie Leresche, Régis C. Lambert

**Affiliations:** ^1^Neuroscience Division, School of Biosciences, Cardiff UniversityCardiff, UK; ^2^Lyon Neuroscience Research Center, Centre National de la Recherche Scientifique UMR 5292Lyon, France; ^3^Lyon Neuroscience Research Center, Institut National de la Santé et de la Recherche Médicale U1028Lyon, France; ^4^Faculté de Médecine, Université Claude BernardLyon, France; ^5^Sorbonne Universités, UPMC Université Paris 06, UM 119, Neuroscience Paris SeineParis, France; ^6^Centre National de la Recherche Scientifique, UMR 8246, Neuroscience Paris SeineParis, France; ^7^Institut National de la Santé et de la Recherche Médicale, U1130, Neuroscience Paris SeineParis, France; ^8^Department of Physiology and Biochemistry, University of MaltaMsida, Malta

**Keywords:** thalamus, sleep slow wave, delta waves, T-type calcium channels, bifurcation, computational modeling

## Abstract

During non-REM sleep the EEG shows characteristics waves that are generated by the dynamic interactions between cortical and thalamic oscillators. In thalamic neurons, low-threshold T-type Ca^2+^ channels play a pivotal role in almost every type of neuronal oscillations, including slow (< 1 Hz) waves, sleep spindles and delta waves. The transient opening of T channels gives rise to the low threshold spikes (LTSs), and associated high frequency bursts of action potentials, that are characteristically present during sleep spindles and delta waves, whereas the persistent opening of a small fraction of T channels, (i.e., I_Twindow_) is responsible for the membrane potential bistability underlying sleep slow oscillations. Surprisingly thalamocortical (TC) neurons express a very high density of T channels that largely exceed the amount required to generate LTSs and therefore, to support certain, if not all, sleep oscillations. Here, to clarify the relationship between T current density and sleep oscillations, we systematically investigated the impact of the T conductance level on the intrinsic rhythmic activities generated in TC neurons, combining *in vitro* experiments and TC neuron simulation. Using bifurcation analysis, we provide insights into the dynamical processes taking place at the transition between slow and delta oscillations. Our results show that although stable delta oscillations can be evoked with minimal T conductance, the full range of slow oscillation patterns, including groups of delta oscillations separated by *Up* states (“grouped-delta slow waves”) requires a high density of T channels. Moreover, high levels of T conductance ensure the robustness of different types of slow oscillations.

## Introduction

Sleep is characterized by the regular appearance of stereotyped sequences of EEG waves (Achermann and Borbely, 1997; Steriade, 2006; Crunelli et al., 2014) that are generated by the dynamic interaction between, and require the integrity of both cortical and thalamic oscillators (Steriade et al., 1993b; Crunelli and Hughes, 2010; David et al., 2013; Lemieux et al., 2014). The various cellular activities that are expressed by thalamocortical (TC) neurons during sleep oscillations tightly depend on the expression of low-threshold T-type Ca^2+^ channels (T channels; Leresche et al., 1991; Williams et al., 1997a; Crunelli et al., 2014). In fact, while these channels are almost fully inactivated in the range of membrane potentials associated to the wake state (but see Lambert et al., 2014), during non-REM sleep the progressive reduction in the depolarizing tone exerted by modulatory afferents onto both cortical and thalamic neurons (McCormick, 1992) allows T channel de-inactivation. As a consequence, the recruitment of de-inactivated T channels generates large inward currents resulting in transient depolarizations, called low-threshold spike (LTS). Thus, rhythmic LTSs, often crowned by bursts of high-frequency (>200 Hz) action potentials, are present in TC neurons during sleep spindles (7–14 Hz; Steriade et al., 1993b; Contreras and Steriade, 1996; David et al., 2013) and delta waves (0.5–4 Hz; Steriade et al., 1993a), and an LTS is almost invariably present at the start of each *Up* state of sleep slow oscillations in TC neurons (**Figure 2A**; Steriade et al., 1993a). *Up* states interspersed with periods of hyperpolarization (i.e., *Down* states) are the thalamic cellular hallmarks of sleep slow (< 1 Hz) waves (**Figure 2A**). Moreover, slow waves group together periods of sleep spindle and delta waves (Steriade, 2006), and these periods of delta oscillations that are visible during the *Down* state of the cellular counterpart of sleep slow waves in TC neurons have been named “grouped-delta slow waves” (**Figure 2A**; Steriade et al., 1993a; Hughes et al., 2002; Crunelli et al., 2015). Importantly, the interaction of the leak current with a small number of de-inactivated T channels opening with a low (but non-zero) probability in a narrow range of membrane potentials around −60 mV (i.e., I_Twindow_; Perez-Reyes, 2003; Dreyfus et al., 2010) is necessary for the generation of the membrane potential bistability that in TC neuron underlies the expression of the *Up* and *Down* state dynamics of sleep slow waves (Williams et al., 1997a; Toth et al., 1998; Hughes et al., 2002; Dreyfus et al., 2010).

Despite these key roles for T channels in sleep waves, it is still not known how the density of the T-type Ca^2+^ current (I_T_) affects each sleep oscillation. We previously demonstrated that robust LTSs can be evoked even when up to 70% of the T channel population is pharmacologically blocked (Dreyfus et al., 2010), suggesting that the high T channel expression that is present in TC neurons is not required for LTS generation during delta and slow oscillations. A high T channel expression in TC neurons, however, may be crucial to provide a level of I_Twindow_ sufficient for the generation of the *UP* and *Down* state dynamics underlying slow oscillations in this type of thalamic neurons.

Here, using both *in vitro* experiments and TC neuron simulation, we systematically investigated the impact of the T conductance level on the various sleep oscillations intrinsically generated in TC neurons. Since I_T_ can be controlled by various modulatory mechanisms (Lambert et al., 2006; Huc et al., 2009), we also investigated the effects of the ATP- and voltage-dependent regulation that potentiates the amplitude of I_T_ in sensory TC neurons (Leresche et al., 2004). Our results show that although stable delta oscillations can be evoked with minimal T conductance, the full range of slow oscillation patterns, including simple *Up* and *Down* state transitions and the more complex “grouped-delta slow waves,” requires a high density of T channels or a potentiation of the current. Moreover, high levels of I_T_ ensure the robustness of different slow wave oscillations over a larger range of leak conductance values.

## Materials and methods

### Slice preparation and recordings

All procedures involving experimental animals were carried out in accordance with the UK Animals (Scientific Procedure) Act, 1986 and Cardiff Ethical Review Committee guidelines. Thalamic slices from a 3-year old cat were prepared as described previously (Hughes et al., 2002). Briefly, the cat was deeply anesthetized with a mixture of O2 and NO2(2:1) and 5% isoflurane, a wide craniotomy was performed to remove the brain and coronal slices of the thalamus (300–400 μm) that contain the dorsal lateral geniculate nucleus (LGN), were prepared and incubated at 35°C for 1 h before being maintained at room temperature. For recording, slices were perfused with a warmed (35 ± 1°C) continuously oxygenated (95% O_2_, 5% CO_2_) artificial CSF (ACSF) containing the following (in mM): 134 NaCl, 2 KCl, 1.25 KH_2_PO_4_, 1 MgSO_4_, 2 CaCl_2_, 16 NaHCO_3_, and 10 glucose.

Intracellular recordings, using the current clamp technique, were performed with standard-wall glass microelectrodes filled with 1 M potassium acetate (resistance, 80–120 MOhm) and connected to an Axoclamp-2A amplifier (Molecular Devices, Sunnyvale, CA) operating in bridge mode. Membrane potentials were digitized at 25 kHz using pClamp 9 (Molecular Devices). All recordings in the LGN were obtained from lamina A. Impaled cells were identified as TC neurons using established criteria (Pirchio et al., 1997; Turner et al., 1997). Sleep oscillations (including slow oscillations < 1 Hz) were induced by bath application of 50 μM (±)-1-aminocyclopentane-*trans*-1,3-dicarboxylic acid (*trans*-ACPD) followed by changes in steady-state current injections to allow neurons to express different slow oscillations, as previously shown (Hughes et al., 2004). SR95531 (gabazine, 10 μM), CGP54626 (20 μM), D-APV (50 μM), and CNQX (10 μM) were included in the bath solution to block both GABA-A and GABA-B as well as NMDA and AMPA glutamatergic synaptic inputs onto TC neurons, respectively. The T channel antagonist, TTA-P2 (kindly provided by Merck Inc, USA), was made up as a 10 mM stock solution in dimethylsulfoxide and kept at −20°C until use at a final concentration of 500 nM.

### Simulations

All simulations were performed using the Matlab based programs (Mathworks, Natick, MA) or xppaut continuation application developed by Ermentrout (2002), and were run with a fixed time step of 0.02 ms using the Euler integration method. For simulations, the system was initiated at a point close to the *Up* state and the simulation results were analyzed only after stabilization of the simulation result (i.e., 50 s after the start of the simulation).

The single-compartment TC neuron model based on (Williams et al., 1997a; Hughes et al., 2002), expressed the essential physiological properties of these neurons (**Figure 2**). Ionic currents were simulated following Hodgkin-Huxley formalism.

The membrane potential (V) was described by the following equation:

$$C_{m}dV/dt=-I_{Leak}-I_{T}-I_{TP}-I_{h}-I_{CAN}-I_{\text{Na}}-I_{Kir,}$$

where Cm (50 pF) is the membrane capacitance, I_Leak_ is a potassium leak current (reversal potential = −95 mV), I_T_ is the T current, I_TP_ is the potentiated component of the T current, I_h_ is the hyperpolarization-activated nonspecific cationic current, I_CAN_ is the Ca^2+^ activated non-selective cation current, I_Na_ is the voltage-dependent Na^+^ current and I_Kir_ is K^+^ current which includes the inward and delayed rectifier components. All current units are pA. Each current was simulated as follows:

I_T_:

$$I_{T}=g_{T}\cdot m^{3}(V)\cdot h(V)\cdot (V(t)-E_{T}),$$

where g_T_ is the maximal conductance and E_T_ = 180 mV is the reversal potential for Ca^2+^ flux. m and h are activation and inactivation variables, respectively, which are defined as follows:

$$\begin{array}{l} \;m_{\infty ,T}=\frac{1}{1+\exp\left(-\frac{v+63}{7.8}\right)},\\ \;\tau_{m,T}=0.612+\frac{1}{\exp\left(\frac{V+16.8}{18.2}\right)+exp\left(-\frac{V+131.6}{16.7}\right)}\\ \;h_{\infty ,T}=\frac{1}{1+\exp\left(\frac{v+83.5}{6.3}\right)},\\ \text{if V}<-80\;\tau_{h,T}=\exp\left(\frac{V+467}{66.6}\right)\\ \text{otherwise }\tau_{h,T}=\left[28+\exp\left(\frac{V+21.88}{10.2}\right)\right] \end{array}$$

I_TP_:

The potentiated component of the T current was modeled by multiplying the T current by a voltage-dependent coefficient P representing the fraction of phosphorylated (“potentiated”) channels (see Leresche et al., 2004 for details)

$$I_{TP}=g_{TP}\cdot m^{3}(V)\cdot h(V)\cdot P(V)\cdot (V(t)-E_{T})$$

**Figure 1:**
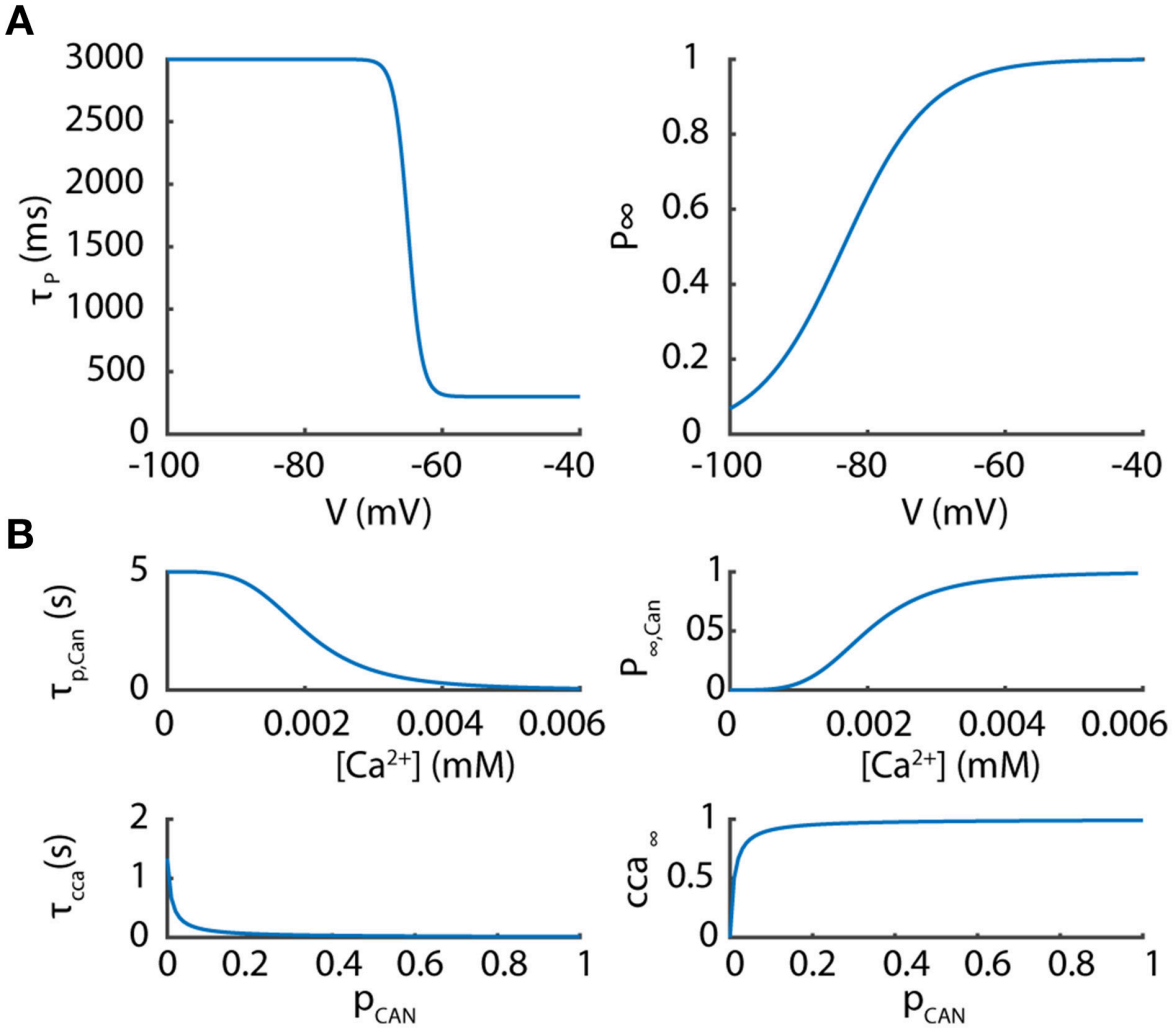
**Parameters of the potentiated component of the T current and the Ca^2+^ activated non-selective cation current. (A)** The potentiated component of the T current is controlled by a voltage-dependent coefficient P whose kinetics and steady values are presented according to membrane potentials. **(B)** The Ca^2+^ activated non-selective cation channel (CAN) is controlled by two variables p_*CAN*_and cca depending upon Ca^2+^ concentration as plotted.

The voltage dependence of P is related to the steady-state inactivation of I_T_ as followed (Figure 1A):

$$P_{\infty ,T}=1-h_{\infty ,T}\;\tau_{P,T}=3000-\frac{2700}{(1+\exp\left(-\left(V+65\right)\right)}$$

I_h_:

$$I_{h}=g_{h}\cdot m^{3}(V)\cdot (V(t)-E_{h})$$

gh = 8 nS

$$\begin{array}{l} \;m_{\infty }{,}_{h}=\frac{1}{1+\exp(\frac{v+75}{5.5})},\text{if V}<-77.57,\\ \tau_{h}=\frac{120819.5}{\exp(-0.0614\cdot V)}\text{ otherwise }\tau_{h}=\frac{29.54}{\exp(0.0458\cdot V)} \end{array}$$

I_CAN_ (Figure 1B):

$$I_{CAN}=g_{CAN}\cdot cca\cdot (V(t)-E_{CAN})$$

g_CAN_ = 12 nS

$$\begin{array}{l} cca_{\infty ,CAN}=\frac{a_{cca}}{a_{cca}+b_{cca}}\;\tau_{cca,CAN}=\frac{1}{a_{cca}+b_{cca}}\\ \;a_{cca}=0.075\cdot p_{CAN}\;b_{cca}=0.00075\\ p_{\infty ,CAN}=\frac{1.25\cdot 10^{7}\cdot {\left[Ca^{2+}\right]}^{4}}{1.25\cdot 10^{7}\cdot {\left[Ca^{2+}\right]}^{4}+0.2}\;\tau_{p,CAN}=\frac{1}{1.25\cdot 10^{7}\cdot {\left[Ca^{2+}\right]}^{4}+0.2} \end{array}$$

The calcium concentration ([Ca^2+^], in mM) is governed by the Ca^2+^ influx through T channels and a Ca^2+^ pump that controls intracellular Ca^2+^ levels.

$$\left[Ca^{2+}\right]=-(I_{T}+I_{TP})\frac{0.0052}{Area\cdot Depth}-5\cdot \left[Ca^{2+}\right]$$

where Area is 5000 μm^2^ and Depth is 0.1 μm.

I_Na_:

$$\begin{array}{l} I_{Na}=g_{Na}m{\left(V\right)}^{3}\cdot h(V)\cdot (V-E_{Na+})\\ {\text{g}}_{Na}=1320nS\\ a_{m,Na}=0.32\;\frac{-V-49.3}{\left(e^{(-V-49.3)/4}-1\right)}\\ b_{m,Na}=0.28\frac{-V-22.3}{1-e^{(-V-22.3)/5})}\\ m_{\infty ,Na}=\frac{a_{m,Na}}{a_{m,Na}+b_{m,Na}}\;\tau_{m,Na}=\frac{1}{a_{m,Na}+b_{m,Na}}\\ a_{h,Na}=0.128\;e^{\frac{-V-45.4}{18}}\;b_{h,Na}=\frac{4}{e^{\frac{-V-22.4}{5}+1}}\\ h_{\infty ,Na}=\frac{a_{h,Na}}{a_{h,Na}+b_{h,Na}}\;\tau_{h,Na}=\frac{1}{a_{h,Na}+b_{h,Na}} \end{array}$$

I_Kir_:

$$\begin{array}{l} I_{Kir}=g_{K}\cdot n^{4}(V)\cdot (V-E_{K})\\ {\text{g}}_{\text{K}}=600\text{nS}\\ a_{n,K}=9.93\cdot 0.016\cdot \frac{-V-57.2+35.1/9.93}{\left(e^{(-V-57.2+35.1/9.93)/5}-1\right)}\\ b_{n,K}=0.25\cdot 9.93\cdot e^{\frac{-V-57.2+20/9.93}{40}}\\ n_{\infty ,K}=\frac{a_{n,K}}{a_{n,K}+b_{n,K}}\;\tau_{n,K}=\frac{1}{a_{n,K}+b_{n,K}} \end{array}$$

### Data analysis

Numerical integrations of the equations without g_Na_ were performed with the software package XXPAUT (Ermentrout, 2002) to compute the periodic and steady-state solutions as a function of a given parameter (either g_Leak_or g_T_). The orbits (or periodic solutions) were detected by continuation of the equation system i.e., by computing the equilibrium solutions of the differential equations of the membrane potential and of other variables by the forward and backward temporal integration of these equations starting from the bifurcation fixed points with Xppaut (http://www.math.pitt.edu/~bard/xpp/xpp.html). The bifurcation parameter (g_Leak_) was varied on adaptative step size between 0.0001 and 0.1 nS and a discretization interval number for periodic orbit of 50. g_Na_ was not used on a first approximation as this fast component easily prevents the system from converging to a stable orbit solution on a slow temporal scale. Stable solutions found without g_Na_ were nonetheless confirmed or infirmed in the system that included g_Na_ in the following steps of the analysis. For *Up* and *Down* state detection, the membrane potential was down-sampled at 1 kHz. *Up* states were defined as the proportion of simulated time where the membrane potential was >−65 mV. An *Up* state episode during slow oscillation was defined as a finite temporal continuous sequence during which the membrane potential remained >−65 mV for more than 500 ms. A *Down* state during slow oscillation was defined as a continuous temporal sequence where the membrane potential remained below the −65 mV threshold. The average membrane potential during an *Up* state was estimated by averaging all membrane potential values belonging to the *Up* state. The number of LTSs per slow oscillation was estimated as the number of *Down* states (which always precede a LTS) divided by the number of *Up* states. Slow oscillation frequency was estimated by averaging instantaneous frequencies measured for each slow oscillation cycle that was defined as starting and finishing with the LTS that is invariably present at the start of each *Up* state.

## Results

**Figure 2:**
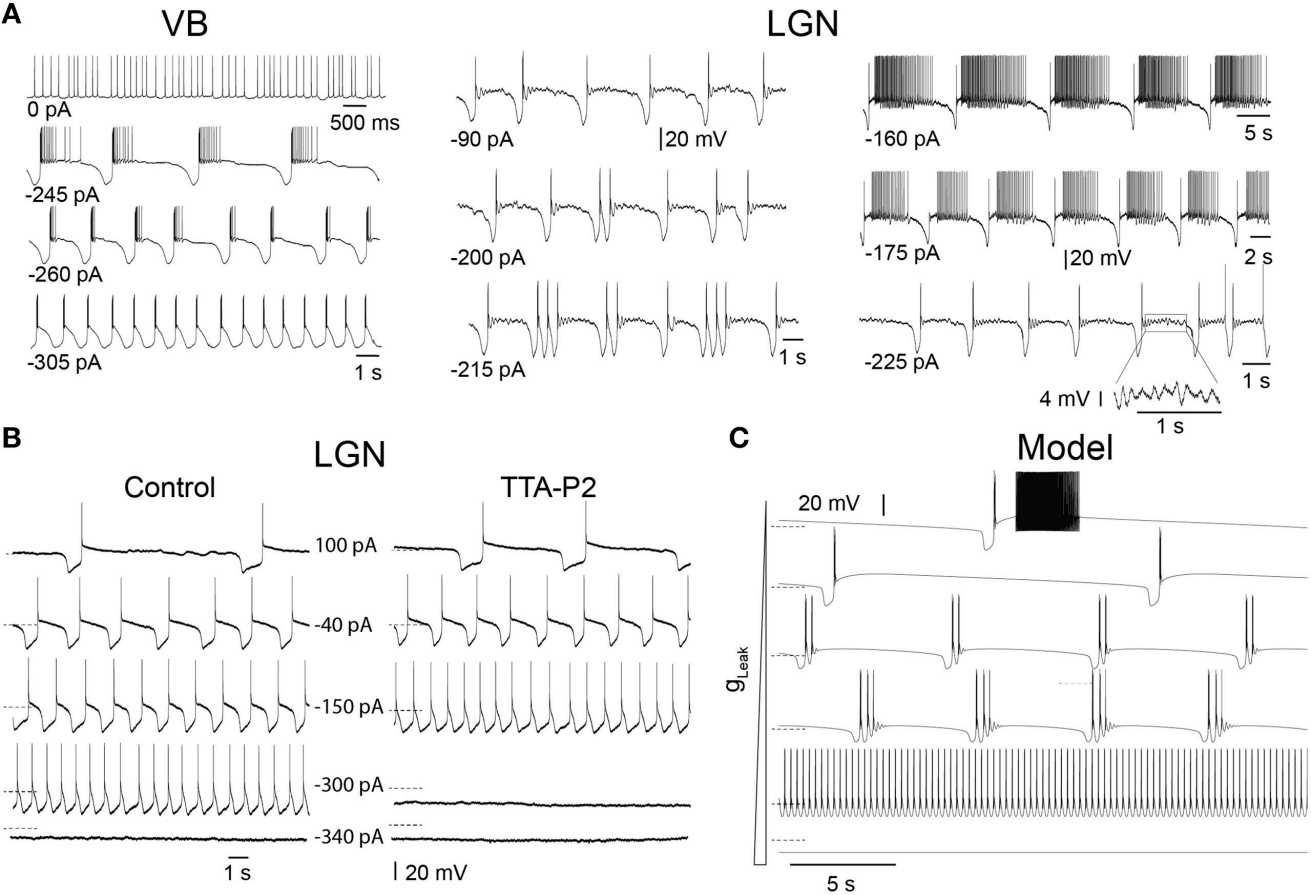
**Different intrinsic oscillations in TC neurons and the effect of low doses of TTA-P2. (A)** Typical membrane potential oscillations recorded *in vitro* in a ventrobasal nucleus (VB) neuron and two lateral geniculate nucleus (LGN) neurons in response to injection of steady hyperpolarizing current of increasing amplitude in the presence of *tran*s-ACPD (adapted, with permission from Zhu et al. (2006). With little current injection, oscillations exhibited *Up* states associated to periods of tonic firing. Increasing the hyperpolarizing current induced slow oscillations comprising quiescent *Up* states followed by isolated LTSs or short episode of delta oscillations. In the VB neuron, continuous delta oscillation was observed with large current injection. Note also the periods of small amplitude, 5–7 Hz oscillations during *Up* states in the LGN neurons (see enlargement of a section of the bottom right trace). **(B)** Membrane potential oscillations recorded *in vitro* in a cat LGN TC neuron in the continuous presence of *tran*s-ACPD before and during perfusion of the slice with the selective T channel blocker TTA-P2. The reduction in I_T_ leads to a narrowing of the range of hyperpolarizing currents that triggers slow oscillations. **(C)** Membrane potential oscillations in a model of TC neuron. As observed in experiments, enhancing the hyperpolarizing drive by increasing g_Leak_ values from 1.5 to 4.2 nS induced (from top to bottom) a transition from slowly alternating *Up* and *Down* states to “group-delta slow waves,” continuous delta oscillations and stable *Down* state. Dotted lines: −65 mV.

In slices, TC neurons of sensory (lateral and medial geniculate, VB), motor (ventrolateral), and intralaminar (centrolateral) thalamic nuclei recorded in the presence of *trans*-ACPD exhibit stereotypical firing patterns and oscillations when submitted to steady hyperpolarizing currents of increasing amplitudes, as we previously described (Hughes et al., 2002; Zhu et al., 2006; Crunelli et al., 2012, 2014): from stable *UP* states, at times showing tonic firing, to slow *Up* and *Down* state oscillations, “grouped-delta slow waves” (i.e., slow oscillations with delta oscillations during the *DOWN* state), pure delta oscillations (1–4 Hz) and stable silent *DOWN* states (Figure 2A). These activities result from the interplay of intrinsic TC neuron conductances, including the T-type Ca^2+^ current (I_T_), with both its transient and window (I_Twindow_) components, the hyperpolarization activated Na^+^-K^+^ current (I_h_), the Ca^2+^ activated non-selective cation current (I_CAN_), the inward rectifying potassium current (I_Kir_) and the leak K^+^ current (I_leak_) (Williams et al., 1997b; Hughes et al., 2002). In order to investigate how I_T_ density affects the expression of these various oscillations, we compared in LGN TC neurons the range of injected steady hyperpolarizing current required to observe the distinct patterns of oscillations in control conditions and when I_T_ was partially blocked by the selective antagonist TTA-P2 (Dreyfus et al., 2010). As shown in Figure 2B, the range of steady hyperpolarizing currents where slow oscillations could occur under control condition (355 ± 31 pA, *n* = 5) was clearly smaller in the presence of TTA-P2 (198 ± 28 pA, *n* = 5), indicating that a reduction in I_T_ drastically weakens the generation of the slow oscillation.

**Figure 3:**
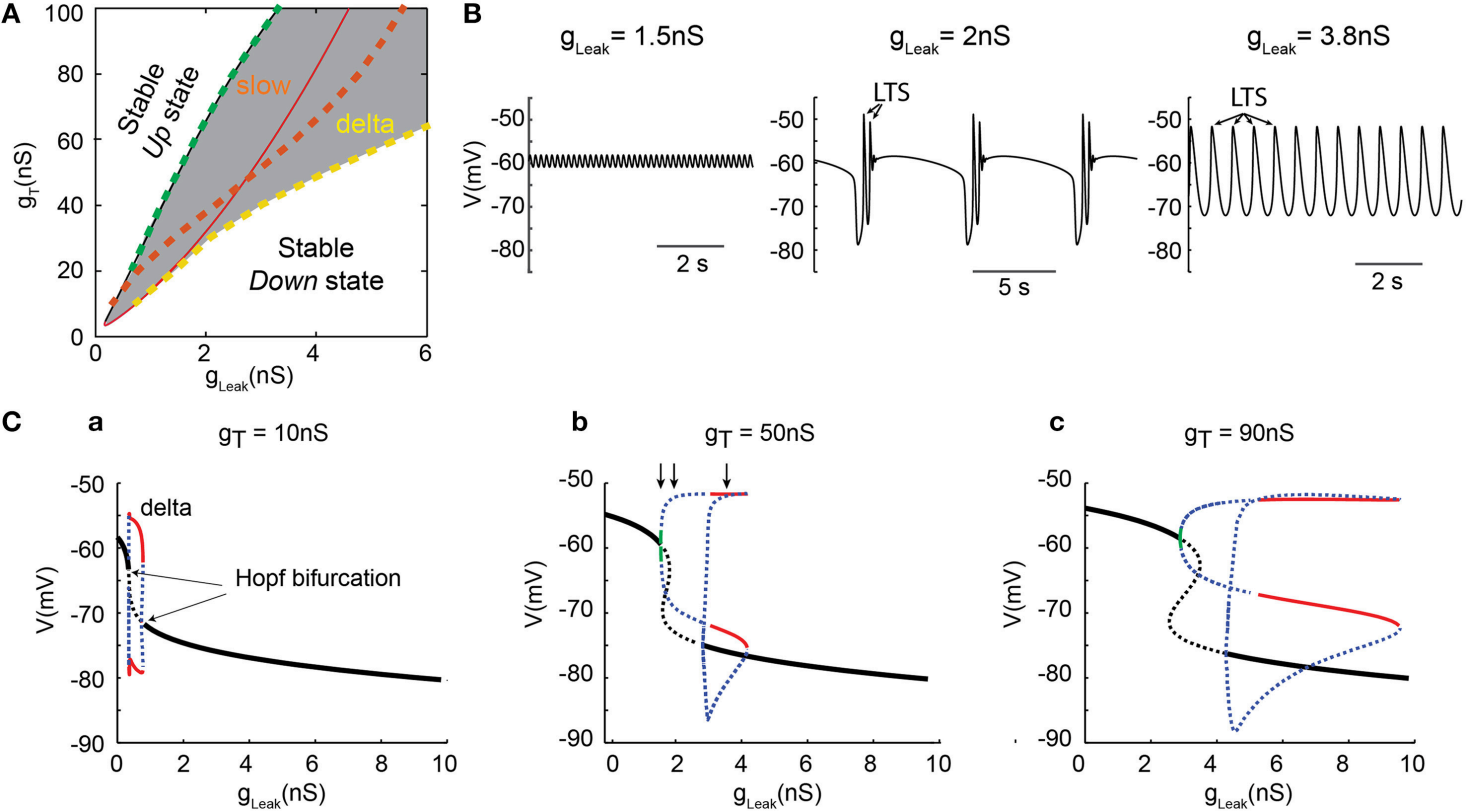
**Dynamical analysis of different oscillations in the TC model cell. (A)** Two-parameter bifurcation diagram indicating the lines of bifurcation of the system in theg_, Leak−_ g_T_ plane: (*i*) black line (almost overlapping with the dashed green line), the (supercritical Hopf) bifurcations from the stable *Up* states (left side) to oscillatory regimes (right side); (*ii*) dashed green line, frontier from the small amplitude 6 Hz oscillations to slow oscillations (right side); (*iii*) red line, the (subcritical Hopf) bifurcations from oscillatory regime (left side) to stable *Down* state (right side); *iv*) dashed orange line, bifurcations from the slow oscillation regime (left side) to continuous delta oscillation (right side); (*v*) dashed yellow line, limit of continuous delta oscillation. The gray zone indicates the domains of oscillations. **(B)** Example of small amplitude 6 Hz oscillations (left), slow oscillations (middle) and delta oscillations (right) observed in the model for g_T_ = 50 nS with g_Leak_, = 1.5, 2, and 3.8 nS, respectively (corresponding to the three vertical arrows in Cb, respectively). Some of the LTSs are indicated by arrows. **(C)** One-parameter (g_Leak_) bifurcation diagrams for 3 increasing values of g_T_. Maximum and minimum membrane potential values (Vm) of small amplitude 6 Hz oscillations (green line), continuous delta oscillations (red lines), unstable orbits (blue dashed lines), and fixed-point equilibria (black lines) for stable *Up* and *Down* states. The dashed black lines indicate the unstable static equilibria. As g_T_ increases, the range of g_Leak_ that allows delta and slow oscillations is drastically increased. These analyses were performed without g_Na_ to simplify computation of the bifurcation diagrams.

To thoroughly analyze the relationship between the T conductance and the ability of TC neuron to generate various sleep-related oscillations, we constructed a minimal single compartment model of a TC neuron that, upon g_Leak_ variation, satisfactorily reproduced the activities observed *in vitro* in response to different steady hyperpolarizing currents (Figure 2C). Although not strictly equivalent, we chose to vary g_Leak_ instead of simulating a hyperpolarizing current injection in order to mimic the natural changes observed across various sleep stages. Using the bifurcation analysis of this dynamic system (without I_Na_ to facilitate analysis, see Materials and Methods), we first calculated the extent of stable *Up* and *Down* states as a function of g_T_ and g_Leak_. As shown in Figure 3A, increasing g_T_ favors a stable *Up* state and larger g_Leak_ values are required to switch the system to a stable *DOWN* state. As already mentioned, I_Twindow_ contributes to the resting membrane potential around −60mV (Dreyfus et al., 2010). Since departure from the stable *Up* state occurs around this potential, a stronger I_Leak_ is required to counteract the depolarizing drive resulting from a large g_T_ and a consequently greater I_Twindow_. The graph also shows the presence of a region of membrane potential instability (delineated by the green and yellow dashed lines in Figure 3A) which occurs for a range of g_Leak_ and g_T_ values. This area of instability can be associated to particular oscillatory dynamics: slow oscillations and continuous delta oscillations (Figure 3B). As already observed experimentally (see Figure 9 in Soltesz and Crunelli, 1992), for some g_T_ values where the system has a subcritical Hopf bifurcation point (Wang and Rinzel, 2002; Amarillo et al., 2015), oscillatory regimes and a stable *Down* state can theoretically occur in the same g_Leak_ domain.

For a small g_T_ (Figure 3Ca), the one-dimension bifurcation diagram of the model system as a function of g_Leak_ remains simple with departure from the stable *Up* or *Down* states involving Hopf bifurcations. The stable periodic orbits correspond to pure delta oscillations (red lines in Figure 3Ca) that do not overlap with the regions where stable *Up* or *Down* states exist. This indicates that small g_T_ values allow only 3 robust exclusive activity patterns in TC neurons: stable *Up* state, pure delta oscillations and stable *Down* state. However, when g_T_ is increased, the bifurcation diagram becomes more complex (Figures 3Cb,c). At departure from the stable *Up*-state, small periodic orbits involving membrane potential oscillations of a few millivolts in amplitude (Figure 3B left) at 6 Hz (or higher frequency) are present for a very narrow range of g_Leak_ (green line in Figures 3Cb,c). Such low-amplitude oscillations that occur close to −60 mV are consistently present in our simulations. Although these oscillations cannot be easily related to any physiologically defined membrane potential waveform of TC neurons, they resemble oscillations that occasionally appear in the *Up* state of slow oscillations in these neurons (Figure 2A; see Hughes et al., 2002; Zhu et al., 2006), and have been suggested to represent the intrinsic dynamic contribution of TC neurons to synaptically generated spindle oscillations (Wang, 1994). As g_Leak_ further increases, unstable orbit cycles (blue dots in Figures 3Cb,c), corresponding to the complex “grouped-delta slow waves” (Figure 3B middle), occur for a large range of g_Leak_ before the stable periodic orbits corresponding to pure delta oscillations (Figure 3B right) could develop (red lines in Figures 3Cb,c). Therefore, although delta oscillations are already present with small g_T_ values, only a larger g_T_ allows the occurrence of the full dynamics observed in TC neurons, including “grouped-delta slow waves.”

**Figure 4:**
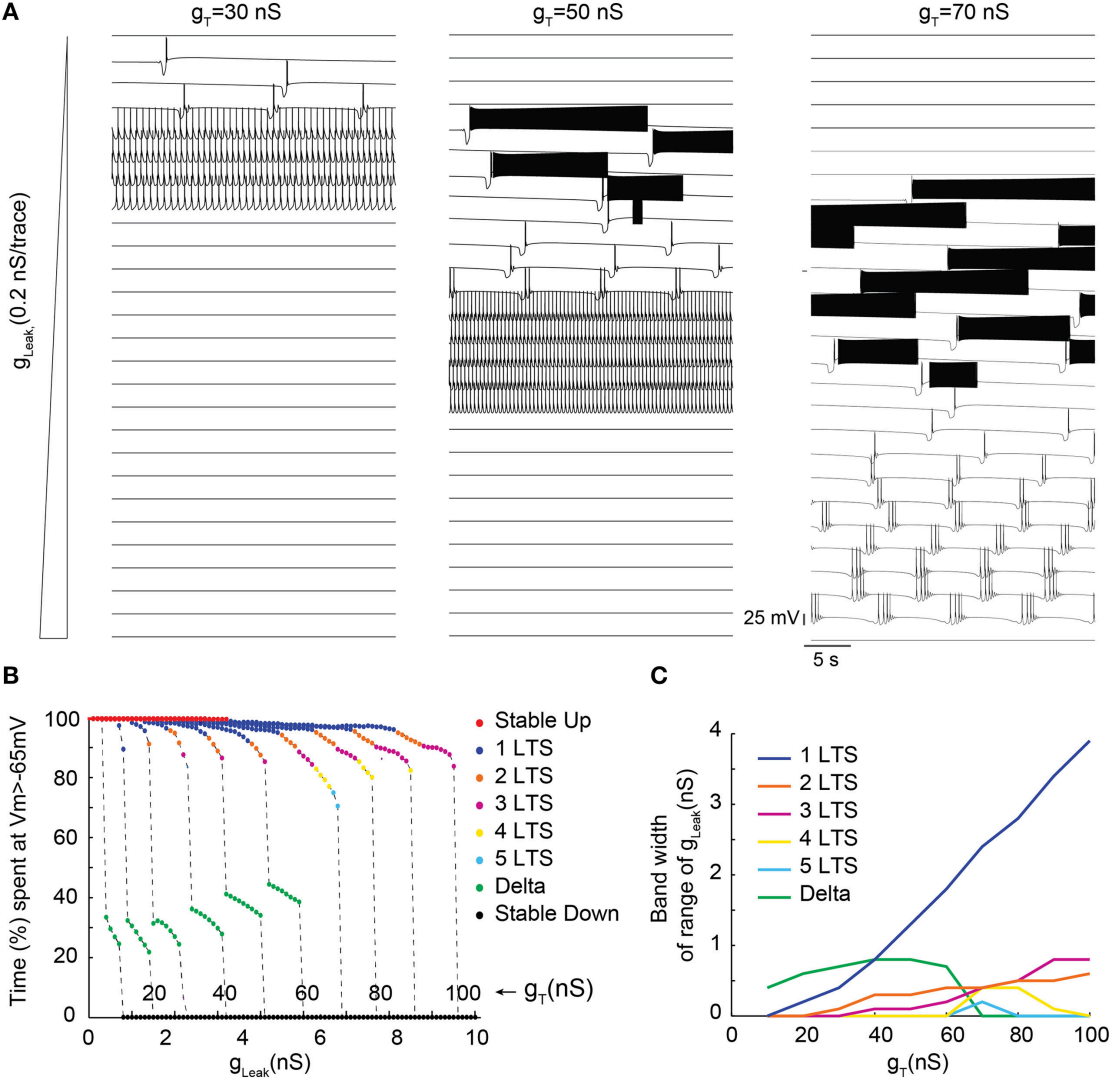
**Membrane potential dynamics as a function of g_Leak_ of TC model cells with increasing g_T_**. **(A)** Examples of membrane potential dynamics for g_T_ = 30, 50, and 70 nS and increasing values of g_Leak_ (from top to bottom). **(B)** Proportion of time spent at depolarized membrane potential (Vm >−65 mV) as a function of g_Leak_ for TC model cells with increasing g_T_ values (from 10 to 100 nS). Dynamic regimes are categorized by color coding according to the number of LTSs per slow oscillation period. **(C)** Range of g_Leak_, in which a given dynamic regime is observed as a function of g_T_.

**Figure 5:**
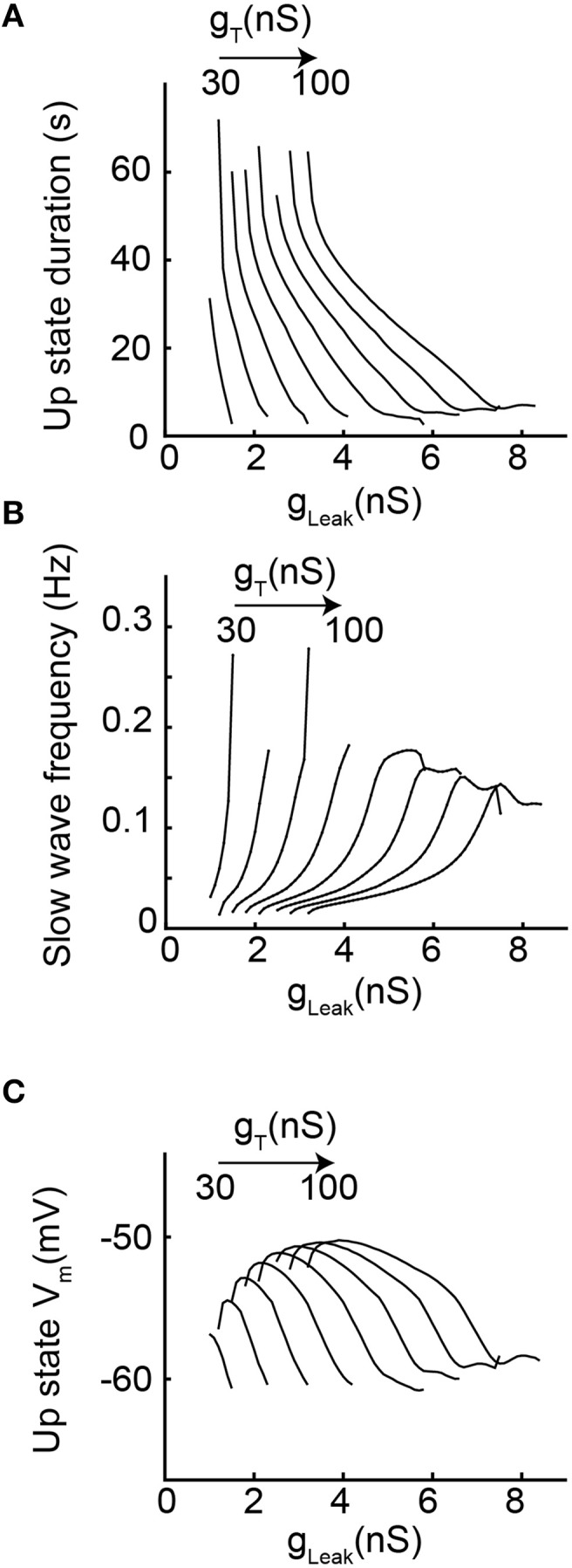
**Slow oscillation properties as a function of increasing g_T_ and g_Leak_. (A)** Each line represents the duration of *Up* state episodes during slow oscillation as a function of g_Leak_, for a given g_T_ (from 20 to 100 nS). **(B)** Slow oscillation frequency for the same data set. **(C)** *Up* state average membrane potential values (see text and Materials and Methods for further details).

In order to more precisely describe the different slow wave patterns present for a given g_T_ value, simulations were then run while systematically varying g_T_ and g_Leak_ (in the presence of g_Na_; Figure 4). Confirming the conclusions of the bifurcation diagrams, analysis of the membrane potential dynamics as a function of g_Leak_ indicates that only delta oscillations occur for the smallest g_T_ value (10 nS, Figures 4B,C). When g_T_ is increased, in addition to continuous delta oscillations, slow oscillations with *Up* states that always start with a LTS are observed in a narrow range of g_Leak_ (Figures 4A–C). For larger values of g_T_, “grouped-delta slow waves” are present and this firing pattern can be observed in a large range of g_Leak_ values that expands as g_T_increases (Figures 4A–C). Further quantification of the slow oscillation parameters indicates that for g_T_ values associated with a robust slow oscillation pattern (g_T_≥ 30 nS), the ranges of *Up*-state duration and slow wave frequencies remain stable (Figures 5A,B), but the maximal value of the *Up* state membrane potential increases proportionally to g_T_ (Figure 5C). These increasingly more depolarized *Up* states may result from both a stronger I_Twindow_ directly linked to the larger g_T_ and the consequently stronger I_CAN_ due to the larger Ca^2+^ entry occurring during the T channel activation that generates the LTS at the beginning of each *Up* state.

Surprisingly, for the highest g_T_ values (>70 nS), although long sequences of “grouped-delta slow waves” are present (Figure 4C), our model cell did not anymore display continuous delta oscillations but abruptly switched from “grouped-delta slow waves” to stable *Down* states when g_Leak_ increased. While the bifurcation diagrams previously calculated for large g_T_ values (Figures 3A,Cc) predicted an increase of the range of g_Leak_ values where stable periodic delta orbits corresponding to continuous delta oscillations may develop, stable *Down* states were the only solutions observed in our simulations (Figure 4C). Such dominance of the stable *Down* state over continuous delta oscillations was due to the presence of action potentials on top of the LTS which elicit large high K^+^ rectifying currents. Indeed, in simulations performed without g_Na_ continuous delta oscillations were observed for some g_Leak_ values (data not shown).

**Figure 6:**
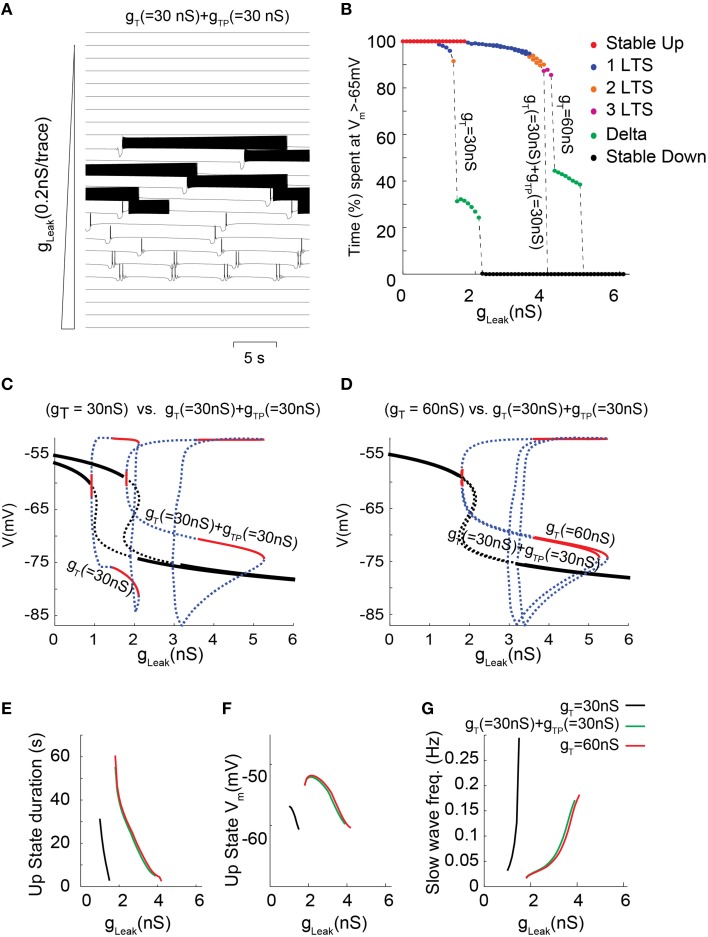
**Membrane potential dynamics as a function of g_Leak_ for a TC model cell that incorporates I_T_ potentiation. (A)** Typical example of membrane potential dynamics for g_T_ = 30 nS + g_TP_ = 30 nS for increasing g_Leak_, values. **(B)** Proportion of time spent at depolarized membrane potential (Vm >−65 mV) as a function of g_Leak_ for TC model cells with g_T_ = 30 nS; g_T_ = 30 nS + g_TP_ = 30 nS; and g_T_ = 60 nS (same color code as in Figure 4B to indicate dynamical regimes). **(C,D)** Effect on the one-parameter (g_Leak_ on x-axis) bifurcation diagrams of either adding a potentiated T conductance (C, g_T_ = 30 nS vs. g_T_ = 30 nS + g_TP_ = 30 nS) or replacing half of the T conductance by a potentiated T conductance (D, g_T_ = 60 nS vs. g_T_ = 30 nS + g_TP_ = 30 nS). Same legend as in Figure 3C. **(E–G)** Slow oscillation characteristics observed in model TC cells with either g_T_ = 30 nS (black); g_T_ = 30 nS + g_TP_ = 30 nS (green) or g_T_ = 60 nS (red). **(E)** Each line represents the duration of *Up* state episodes during slow oscillation as a function of g_Leak_, for a given g_T_ and g_TP_. **(F)** Slow oscillation frequency for the same data set. **(G)** *Up* state average membrane potential values.

Although the prominent I_T_ in TC neurons mainly results from a high channel expression, we previously demonstrated that in neurons of sensory thalamic nuclei, I_T_ amplitude is also transiently potentiated by a phosphorylation (ATP-dependent) mechanism, which exclusively occurs when the channels are inactivated, i.e., it increases with membrane depolarization (Leresche et al., 2004). To study how this additional mechanism that drastically controls the T current amplitude in this population of TC neurons contributes to their firing dynamics a new set of simulations was run where part of the total I_T_ was due to a “potentiated” T conductance (g_TP_). The kinetics, amplitude and voltage-dependence of this g_TP_ mimic the effect and properties of the described phosphorylation mechanism (see Materials and Methods for further details; Leresche et al., 2004). When I_T_ was increased by introducing g_TP_ in the model, a strengthening of the slow oscillation which occurred in a larger range of g_Leak_ values is once again observed (Figure 6C; also compare Figures 6A,B with Figures 4A,B). However, for a given value of T conductance, negligible differences in the bifurcation diagrams are observed when comparing the dynamical behaviors supported either by non-potentiated currents or by a combination of non-potentiated and potentiated currents (Figures 6D–G). Hence, the peculiar biophysical properties of the potentiated T conductance do not significantly modify the membrane potential dynamics of slow oscillations. Nevertheless a close examination of the oscillatory regimes shows that for T conductance values where both “grouped-delta slow waves” and continuous delta developed (Figure 6B, g_T_ 60 nS), the continuous delta disappeared upon introduction of the voltage-dependent potentiation (Figure 6B, g_T_ 30 nS+g_TP_ 30 nS). This suggests that compared to a simple increase in g_T_, this ATP-dependent T channel regulation can selectively enhance the occurrence of slow oscillations of TC neurons at the expenses of delta oscillations.

## Discussion

Since their first development (Rose and Hindmarsh, 1989), TC neuron models have gained in precision and completeness (Destexhe et al., 1998), thus allowing detailed analysis of the dynamical processes that are intrinsic to these neurons (Destexhe and Sejnowski, 2003; Amarillo et al., 2015). Our current model adds to this knowledge by providing for the first time insights into the dynamical processes that take place at the transition between slow and delta oscillations. In particular, our results strongly suggest that the high g_T_ of TC neurons, either due to channel expression or regulation, is not required to generate full-blown LTSs during delta and slow oscillations but is necessary for the generation of the *Up* and *Down* state dynamics underlying the slow oscillation of these neurons (David et al., 2013; Crunelli et al., 2014).

Contrary to the interpretation of the original *in vitro* and *in vivo* studies (see Crunelli et al., 2015), it is now well established that the full expression of slow oscillations requires both cortical and thalamic activities. In particular, combining ensemble recordings of single TC neurons and reverse microdialysis, we recently showed that slow wave frequency is strongly reduced following intrathalamic application of either TTX or TTA-P2 in both anesthetized and naturally sleeping rats (David et al., 2013). In agreement with these data, mice with a Cav3.1 deletion in the thalamus (but not in the cortex) experience frequent arousals during sleep (Anderson et al., 2005), supporting the importance of thalamic T channels in stabilizing sleep rhythms. Moreover, the biophysical mechanisms underlying the conditional thalamic oscillator responsible in TC neurons for the full manifestation of different types of slow oscillation depends on the membrane potential bistability that is created by the interaction between I_Twindow_ and I_Leak_ (Williams et al., 1997a; Toth et al., 1998; Crunelli et al., 2006). TC neurons present a small I_Twindow_ (a few tens of pA; Dreyfus et al., 2010) and any decrease in this current may drastically impact its ability to play a significant physiological role. Our simulations results indicate that the high T channel expression in TC neurons is crucial to generate a large enough I_Twindow_ capable of supporting the *Up* and *Down* states dynamics of slow oscillations over a large range of g_Leak_ values. This was clearly confirmed by our *in vitro* recordings showing that partial block of the I_T_ by TTA-P2 drastically reduces the range of steady hyperpolarizing currents that can generate intrinsic slow oscillations in TC neurons.

In addition, our simulations have indicated that a large g_T_ is also essential for the appearance of “grouped-delta slow waves.” As indicated above, during slow waves the voltage-dependence of I_Twindow_ creates the membrane potential bistability but the rhythmic occurrence of *Up* and *Down* states relies on the dynamics of I_CAN_ (Hughes et al., 2002). Indeed, upon Ca^2+^ entry via the T channels these mixed cationic channels generates a transient depolarizing current that adds to I_Twindow_ to set the membrane potential of the *Up* state. As intracellular Ca^2+^ slowly return to its basal level, the progressive decrease of I_CAN_ reduces this membrane potential up to the point where the stable *Up* state equilibrium disappears and the membrane potential switches to the *Down*-state. With medium g_T_ values, Ca^2+^ entry during LTS is moderate and I_CAN_ activation, together with I_Twindow_, is not strong enough to counteract a strong I_Leak_ and thus to generate the stable equilibrium necessary for an *Up*-state. Consequently, TC neurons go into continuous delta oscillations. However, with higher g_T_ values, Ca^2+^ accumulation after a few delta oscillation cycles is sufficient to maximally activate I_CAN_ and thus set an *Up* state equilibrium that terminates a delta oscillation episode.

Importantly, when our simulations included g_Na_ we did not observe continuous delta oscillations for high or potentiated T channel conductances. Indeed, during natural sleep, thalamic delta oscillations appear to occur mostly in discrete groups during the down state of slow oscillations in both TC and nucleus reticularis thalami neurons (Steriade et al., 1993c; Timofeev and Steriade, 1996) and there is no evidence supporting the presence of continuous delta oscillations in TC neurons *in vivo*. Therefore, one can hypothesize that as suggested in Figure 3A strong T channel expression may prevent the appearance of continuous intrinsic rhythmicity at delta frequency in TC neurons. Interestingly, we previously showed that the maximal amplitude of I_T_ is highly variable across neurons of different thalamic nuclei and even in different TC neurons within a nucleus (Leresche et al., 2004). Therefore, further modeling studies should aim to investigate how this heterogeneity in T channel density among TC neurons interacts with other intrinsic conductance expression such as I_CAN_ or Ih to impact oscillatory dynamics.

Finally, one has to consider that the slow oscillation modeled here is generated intrinsically by single TC neurons (i.e., recorded in the presence of both glutamate and GABA blockers) and thus without the influence of either excitatory cortical and inhibitory inputs. Although this should represent the basic cellular mechanism explaining the conditional role played by the thalamus in sleep slow waves generation (Crunelli and Hughes, 2010), the precise interactions between this intrinsic mechanism and the complex thalamocortical activities (Sheroziya and Timofeev, 2014) remain to be clarified. Along the same line our simulations cannot inform on the firing dynamics of TC neurons during the expression of absence seizures since this abnormal activity requires the integrity of the thalamocortical network (Crunelli and Leresche, 2002). However the present results may help to better understand firing patterns observed during general anesthesia where the EEG mostly includes spindle and delta waves (Franks, 2008). In particular, phase 2 of the maintenance period is characterized by an increase in delta (0–4 Hz) activity (Brown et al., 2010). At clinically relevant concentrations, a number of volatile general anesthetics have been shown to inhibit both recombinant and native T channels (Orestes and Todorovic, 2010; Eckle et al., 2012), a result which was considered at first glance in contradiction with the occurrence of delta oscillations. However, (Eckle et al., 2012) showed that anesthetic doses of isoflurane only inhibits 20 to 60% of I_T_ but induce a marked decrease of I_Twindow_ in TC neurons. In this respect, our simulations clearly indicate that such partial inhibition of I_T_ should indeed favor the occurrence of continuous delta oscillations, at least in TC neurons. In agreement with this view, a partial block of I_T_ by *in vivo* administration of TTA-related compounds also produce sedative effects in behaving mice (Uebele et al., 2009; Kraus et al., 2010), further illustrating the complex relationship between the amount of g_T_ and various sleep-related activities.

## Author contributions

FD, VC, NL, RL contribute to the design of the work, the acquisition, analysis and interpretation of data. FD, VC, NL, RL contribute to the manuscript and approve the final version.

## Funding

The work was supported by the Centre National de la Recherche Scientifique (LIA 528) and the Wellcome Trust (grant 91882).

### Conflict of interest statement

The authors declare that the research was conducted in the absence of any commercial or financial relationships that could be construed as a potential conflict of interest.
